# Considering Harm and Safety in Youth Mental Health: A Call for Attention and Action

**DOI:** 10.1007/s10488-014-0577-x

**Published:** 2014-07-23

**Authors:** Miranda Wolpert, Jessica Deighton, Isobel Fleming, Peter Lachman

**Affiliations:** 1 Evidence Based Practice Unit and CORC, UCL and Anna Freud Centre, 21 Maresfield Gardens, London, NW3 5SU UK; 2 CORC, London, UK; 3 Great Ormond Street Hospital for Children NHS Foundation Trust, London, UK; 4 Evidence Based Practice Unit, UCL and Anna Freud Centre, 21 Maresfield Gardens, London, NW3 5SU UK

**Keywords:** Harm, Safety, Child mental health

## Abstract

The possibility of harm from mental health provision, and in particular harm from youth mental health provision, has been largely overlooked. We contend that if we continue to assume youth mental health services can do no harm, and all that is needed is more services, we continue to risk the possibility that the safety of children and young people is unintentionally compromised. We propose a three level framework for considering harm from youth mental health provision (1. ineffective engagement, 2. ineffective practice and 3. adverse events) and suggest how this framework could be used to support quality improvement in services.

## Introduction

Mental health provision for young people (under the age of 25) is a major area of concern internationally. In both the UK and the US there is increasing recognition of the long term impact of youth mental health problems and the need for a more coordinated response (e.g. Department of Health 2014; Treatment Advocacy Center 2014). In the UK a parliamentary enquiry is considering what is seen as a crisis in mental healthcare provision for youth (Commons Select Committee 2014; Hindley 2014). Discussion of “harm” or “safety” in relation to child mental health has been largely focused on lack of provision (Campion et al., 2013) and the perceived resultant negative impact on clinical outcomes (e.g. Treatment Advocacy Center 2014) or on safeguarding requirements for young people at risk of harming themselves or others (e.g. Treatment Advocacy Center, 2014). What little research there has been on patient safety in mental health has focused on services for adults or has been identified through the Serious Case Review process after a child death and the subsequent recommendations for relevant organizations. Commonality between reviews indicates key features of a lack of clearly agreed definitions or common awareness amongst service providers and a lack of suggested mechanisms to embed in practice (e.g. Brickell et al. 2009; Wachter 2010).

In recent years there has been increasing reference to the possibility of harm from psychological therapy (Hansen et al. 2006; Lambert and Shimokawa 2011) and the need for clinicians to be more aware of the possible negative impact of ineffective therapy (Boisvert and Faust 2006). There has more recently been an additional focus on adverse effects in therapy (e.g. AdEPT: Understanding and Preventing Adverse Effects of Psychological Therapies, 2011–2014, project funded by NIHR Research for Patient Benefit; Lilienfeld 2007) and the start of a debate about potential definitions and parameters of harm from psychotherapeutic treatment (Castonguay et al. 2010; Dimidjian and Hollon 2010). However, this has not yet been rigorously considered in relation to youth mental health.

This is in contrast to the priority given to consideration of “harm” and “safety” across physical healthcare (Ginsburg et al. 2014). In the UK, post the reports on scandals in health care safety (Francis 2013; National Advisory Group on the Safety of Patients in England 2013), the NHS is preoccupied with the improvement of the quality of care and, in particular, safety of patients. Likewise, in the US the move to consider safety across all hospitals continues apace (Meeks et al. 2014). Yet, despite the increased emphasis on safety in physical health and the proclaimed policy priority to promote “parity of esteem” across physical and mental health, there appears to be an absence of attention to measures of safety within mental health, particularly in youth mental health services. For example, in England the two organizations who jointly oversee payment systems across the NHS justified a cut in prices for mental health provision by arguing that mental health services did not have to bear the cost of implementing the safety recommendations made following safety scandals (Lintern 2014).

What is urgently required is a systematic approach to the measurement of harm in youth mental healthcare and the embedding of systems to ensure safety. This requires relatively small changes to the way data are currently collected but major changes in the way that these data are used and conceived. This change could have a massive impact on patient safety and could provide youth mental health services in an environment of continual improvement.

**Fig. 1:**
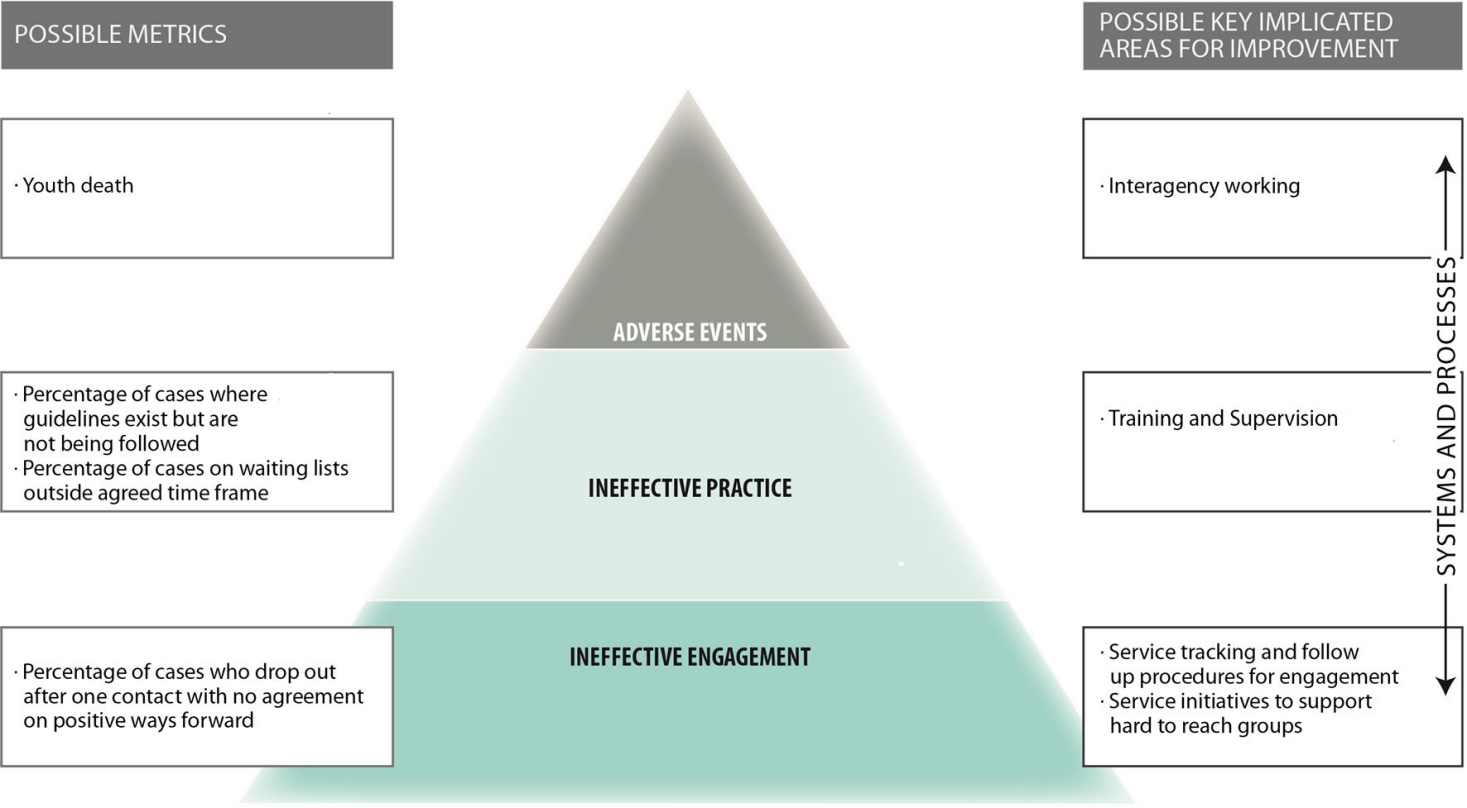
Preliminary framework for consideration of patient safety in CAMHS

Below we present a possible framework that conceptualizes harm in youth mental health as arising at three escalating levels of harm (see Fig. 1). This takes a wider definition of harm than that proposed by Dimidjian and Hollon (2010) in that it includes both harm from ineffective or unhelpful treatment and builds on work in pediatric physical health contexts in the USA and UK (Muething et al. 2012). Because this is a new area we have kept the conceptualization relatively broad but would anticipate further distinctions and refinement over time. The proposed framework focuses on harm and safety *within* healthcare provision rather than harm caused by lack of access in the first place, which has been well documented elsewhere (e.g. Campion et al. 2013). We propose that these metrics would be considered as part of routine data collection relative to that of services with a similar case mix in order to identify outliers and consider unwarranted variation. The metrics will encourage collaborative solution hypothesizing by clinicians, monitoring bodies, funders and service users. These should be used alongside greater consideration of harm being built into research both quantitative and qualitative along the lines suggested by Dimidjian and Hollon (2010).

## Harm Caused by Ineffective Engagement

This level of harm relates to harm caused by young people stopping contact with services before they have received the help they need. Audits from across the world have reported high rates of service users ending treatment prematurely, generally from around 20-40 % (Dejong et al. 2012; Kazdin 2004; Luk et al. 2001). For children under the age of 16, non-attendance is generally a parental or carer decision, and failure to attend may be seen as a withholding of access to treatment by adults (Powell and Appleton 2012).

Engagement also needs to be seen as a two-way process, whereby services as much as families take responsibility for ensuring continued contact, as required. Youth mental health services need to rigorously collect information on dropouts and to put in place systems to benchmark against who drops out and how these children and families are followed up. Families who disengage and move between services are regularly noted in child protection enquiries. Services identified as having unwarranted levels of disengagement compared with other services would need to consider how clinicians are engaging with families, including, for example, implementation of shared decision-making and what follow-up mechanisms for non-attenders are in place.

## Harm Caused by Ineffective Practice

Key to high quality, safe services is the delivery of effective care and measurement of outcomes to ensure positive impact (e.g. Children and Young People’s Improving Access to Psychological Therapies [CYP IAPT] 2014). It is important to note that positive impact does not simply equate to improved functioning post intervention, as this may be no more than the natural course of the underlying problem (Dimidjian and Hollon 2010). Rather it needs to be functioning improved compared to what would have been achieved if no intervention had been provided at all (Wolpert et al. 2013). In England the National Institute for Health and Care Excellence (NICE) has 23 clinical guidelines for treatment in youth mental health. However, evidence from local audits and peer review networks indicates that many youth mental health services are not yet implementing or offering NICE backed treatments (Kelvin et al. 2009). In addition, research indicates that evidence-based treatment may not, in and of itself, lead to positive impact (Garland et al. 2014; Weisz et al. 2006). Perhaps even more worryingly, many (perhaps most) services are not routinely considering the impact of their work (Batty et al. 2013) which has been shown to limit service effectiveness (Lambert et al. 2003). Services need to measure both the implementation of specified packages of care and the impact of these interventions and systems. It is appreciated that inferring causality will be complex and is likely to involve triangulation of data across domains and perspectives (Dimidjian and Hollon 2010; Wolpert et al. 2014). Findings can then be used to inform quality improvement and service change initiatives (The Health Foundation 2014).

## Harm Caused by Adverse Events

There are a number of systems in physical health for registering adverse effects (such as the “yellow card” system in the UK, and post-marketing surveillance systems used by Food and Drug Administration in the US). It may be that we need to develop one for child mental health as a priority action. A first step would need to be stakeholder agreement across clinicians as to what might constitute an adverse event in youth mental health. As Dimidjian and Hollon (2010) note, reaching consensus on what constitutes an adverse event may itself be complex. In the Canadian review of mental health patient safety for adults (Brickell et al. 2009) a range of potential adverse events were suggested for measurement in inpatient contexts including: patient victimization, seclusion and restraint, falls and other patient accidents. Some of these, though not all, apply to the youth outpatient population as well. Serious harm reviews following child deaths have highlighted the impact of poor interagency working in such tragic cases. A Delphi style consultation of youth mental health providers is urgently required to develop agreed indicators for this community for both inpatient and outpatient care. Once agreed, safety responses to unwarranted levels of variation could lead to investigation of team working and key indicators of good and poor practice.

## Conclusion

We believe that the lack of formal consideration of safety in mental health, and in particular youth mental health, needs to be addressed now. We contend that if we continue to assume youth mental health services can do no harm, and all that is needed is more of them, we continue to risk the possibility that the safety of children and young people is unintentionally compromised.
